# WWP1 inhibition suppresses the proliferation of pancreatic cancer cells by regulating the PI3K-AKT pathway

**DOI:** 10.1007/s00535-024-02192-x

**Published:** 2024-12-10

**Authors:** Genso Notoya, Takahiro Kishikawa, Kengo Yasugi, Takuma Iwata, Takahiro Seimiya, Koji Miyabayashi, Ryota Takahashi, Keisuke Yamamoto, Hideaki Ijichi, Motoyuki Otsuka, Mitsuhiro Fujishiro

**Affiliations:** 1 Department of Endoscopy and Endoscopic Surgery, Graduate School of Medicine, The University of Tokyo, Tokyo, 113-8655 Japan; 2 Department of Gastroenterology, Graduate School of Medicine, The University of Tokyo, 7-3-1 Hongo, Bunkyo-ku, Tokyo, 113-8655 Japan; 3 Clinical Nutrition Center, Graduate School of Medicine, The University of Tokyo, Tokyo, 113-8655 Japan

**Keywords:** WWP1, Pancreatic cancer, PTEN, PI3K inhibitor

## Abstract

**Background:**

The proto-oncogene *WWP1* is overexpressed in various cancers and contributes to tumor growth and poor prognosis. Recently, WWP1 inhibition was reported to suppress tumor development and cell proliferation by activating the PTEN function. However, the expression profiles and clinical significance of WWP1 in pancreatic ductal adenocarcinoma (PDAC) tissues remain undetermined. Therefore, this study aimed to evaluate the WWP1 expression in PDAC and investigate the therapeutic potential of WWP1 inhibition.

**Methods:**

Cellular proliferation assays were performed using a doxycycline-inducible shWWP1 expression system. Transcriptome analyses were conducted to identify the altered pathways in *WWP1*-depleted cells. PTEN ubiquitination by WWP1 was confirmed using immunoprecipitation assays. In vivo xenograft and drug screening assays were performed to evaluate the clinical significance of WWP1 inhibition.

**Results:**

WWP1 was significantly upregulated in PDAC tissues and associated with poor prognosis. *WWP1* depletion significantly reduced the proliferation of PDAC cell lines, correlating with the suppression of the PI3K-AKT pathway. Mechanistically, as reported in other cancer types, PTEN is a target of WWP1 in PDAC cells. *PTEN* silencing abrogated the growth-inhibitory effects in *WWP1*-depleted cells, suggesting that the anti-tumor effects of WWP1 inhibition are mediated through PTEN activation. In vivo xenograft studies confirmed that *WWP1* depletion substantially inhibited tumor growth. Moreover, drug screening assays revealed that *WWP1* depletion had an additive effect with the PI3K-AKT pathway inhibitors on hindering tumor growth.

**Conclusion:**

WWP1 inhibition enhances the anti-tumor effects of PI3K-AKT pathway inhibitors through PTEN activation. Thus, WWP1 could be a potential therapeutic target in PDAC.

**Supplementary Information:**

The online version contains supplementary material available at 10.1007/s00535-024-02192-x.

## Introduction

Pancreatic ductal adenocarcinoma (PDAC) is an intractable cancer with an extremely poor prognosis and a 5-year survival rate of 12% [1]. Its associated morbidity and mortality rates continue to increase; it is estimated that it will become the second leading cause of cancer-related deaths in the United States by 2030 [2]. Thus, there is an urgent need to develop novel therapeutic agents for PDAC and elucidate their molecular mechanisms of action to improve the PDAC prognosis.

WWP1, an HECT-type ubiquitin E3-ligase [3], is frequently amplified and mutated in multiple cancers, including hepatocellular carcinoma and prostate and breast cancers. *WWP1* depletion in those cancers demonstrated growth-inhibitory effects in vivo and in vitro [4–6]. Mechanistically, WWP1 regulates the stability and function of several tumor-suppressive proteins by ubiquitinating them, including p27 [7], RNF11 [8], and SMADs [9, 10]. However, no previous reports explored the expression profile of WWP1 in PDAC tissues.

PI3K-AKT pathway activation accelerates carcinogenesis and is associated with drug resistance and poor prognosis in PDAC [11, 12]. Therefore, inhibition of this pathway could potentially have anti-tumor effects [12]. However, the clinical efficacy of PI3K and AKT inhibitors has been limited due to the acquisition of drug resistance and the occurrence of side effects, particularly hyperglycemia [13–15]. Recently, WWP1 was reported to ubiquitinate and inactivate PTEN, which in turn activates the PI3K-AKT pathway and contributes to tumor growth [16, 17]. The deletion or mutation of the key tumor suppressor gene *PTEN* is associated with the development and progression of multiple cancers, especially hepatocellular carcinoma and prostate and breast cancers [18–20]. In PDAC mouse models, *Pten* knockout was suggested to play a crucial role in promoting the development of precancerous lesions and carcinogenesis in the pancreas [21, 22]. However, *PTEN* has not been clinically focused on due to the low frequency of mutations or deletions in human PDAC tissues [23], despite the fact that the PTEN expression levels tend to be downregulated in the tumors [24]. Notably, the main mechanism of PTEN inactivation by WWP1 is mediated through its dissociation from the plasma membrane, where PTEN dephosphorylates PIP_3_ and converts it to PIP_2_, and not through the conventional ubiquitin–proteasome degradation machinery [25]. This indicates that WWP1 inhibition can function as an additional suppressor of the PI3K-AKT pathway through reactivation of PTEN in PDAC, in which PTEN predominantly exists in the wild genotype. Notably, combined treatment with WWP1 and PI3K inhibitors has demonstrated a synergistic anti-tumor effect in breast cancer cells that were resistant to PI3K inhibitors [26].

To date, the expression profiles and clinical significance of WWP1 in PDAC tissues remain unexplored. Therefore, this study aimed to evaluate the WWP1 expression in PDAC and investigate the therapeutic potential of WWP1 inhibition associated with the activation of the PI3K-AKT pathway.

## Methods

### Cell culture

Human pancreatic cancer cell lines BxPC-3, Panc-1, Capan-1, MIAPaCa-2, and AsPC-1, as well as human normal epithelial cell line HPNE, were obtained from the American Type Culture Collection. KP-4 and SUIT-2 were purchased from the Japanese Collection of Research Bioresources. PaTu8988S and PaTu8988T cells were obtained from the DSMZ-German Collection of Microorganisms and Cell Cultures GmbH. The human embryonic kidney cell line HEK293T was purchased from System Biosciences (SBI, Mountain View, CA, USA). BxPC-3, AsPC-1, KP-4, and SUIT-2 cells were cultured in RPMI medium supplemented with 10% FBS. Panc-1, MIAPaCa-2, PaTu8988S, PaTu8988T, and HEK293T cells were cultured in Dulbecco’s Modified Eagle’s medium (DMEM) supplemented with 10% FBS. Capan-1 cells were cultured in Iscove’s Modified Dulbecco’s medium (IMDM) supplemented with 20% FBS. All cells were incubated at 37 °C in an atmosphere of 20% O_2_ and 5% CO_2_.

### Plasmid construction

shRNA oligonucleotides were annealed and cloned into pLKO.1–Puromycin and Tet-pLKO-Puromycin lentiviral vectors (Addgene, Watertown, MA, USA). The sequences used for the respective target genes were as follows:

shScramble: 5′-CCTAAGGTTAAGTCGCCCTCG-3′

shWWP1 #1:5′-ATTGCTTATGAACGCGGCTTT-3′

shWWP1 #2:5′-ACAACACACCTTCATCTCCGT-3′

### Transfection and lentivirus transduction

A lentiviral packaging system (SBI) was used to generate stably expressing cells according to the manufacturer’s protocol. Briefly, the plasmid and pPACK1 packaging plasmid mix were transfected into HEK293T cells using Effectene Transfection Reagent (Qiagen). After 48 h, the viruses were concentrated in the culture medium using PEG-it Reagent (SBI). The centrifuged pellet was resuspended in 1 × PBS, and aliquots were stored at – 80 °C. Cells were infected with the virus using a polybrene reagent (Merck Millipore), followed by selection with 2 µg/mL puromycin for at least 3 days.

### Western blotting

Western blotting was performed as previously described [27]. Briefly, total lysate samples were separated on 12.5% or 5–20% gradient polyacrylamide gels (Fujifilm Wako Pure Chemicals) and transferred onto Immobilon-P membranes (polyvinylidene fluoride; Merck). After blocking with 5% skimmed milk, the membranes were probed with the appropriate primary antibodies diluted in Immunoshot Reagent I (Cosmo Bio) for 16 h at 4 °C. The corresponding HRP-conjugated secondary antibodies (GE HealthCare) were then added. Bound antibodies were visualized using the ImmunoStar LD Reagent (Fujifilm Wako Pure Chemicals). Blot images were obtained using a WSE-6100H LuminoGraph I (ATTO Corporation, Tokyo, Japan). The antibodies used in this study are listed in Table S1.

### Immunoprecipitation

The cells were lysed using RIPA buffer (Fujifilm, Wako Pure Chemicals). Total lysates were precleared using Pierce Protein A/G Magnetic Beads (#88802, Thermo Fisher Scientific) for 15 min at 4 °C, and then immunoprecipitated using anti-PTEN or anti-rabbit IgG antibody (Table S1) bound with Pierce Protein A/G Magnetic Beads overnight at 4 °C. The immunoprecipitates were washed three times using RIPA buffer and eluted with Pierce IgG Elution Buffer (#21004, Thermo Fisher Scientific). Samples were separated on 5–20% gradient polyacrylamide gels (Fujifilm Wako Pure Chemicals) and subjected to western blot analysis.

### Subcellular fractionation

BxPC-3 cells expressing TetOn shWWP1 were treated with or without 1 µg/mL of Dox and cultured for 48 h. Membrane and cytosolic fractions were subsequently isolated using the Subcellular Protein Fraction Kit for Cultured Cells (Thermo Fisher Scientific), following the manufacturer’s protocol.

### Cell proliferation assay

Cell proliferation was evaluated using a crystal violet assay. Cells at the 0, 24, 48, 72, and 96 h time points were fixed using 10% formalin and stained using 0.05% crystal violet. They were then solubilized using 10% acetic acid, and the absorbance was measured using a Multiskan FC Microplate Photometer (Thermo Fisher Scientific).

### Cell cycle analyses

Cells harvested from a 6-well plate were fixed in 70% ice-cold ethanol and incubated for 24 h at − 20 °C. The fixed cells were washed twice using 1 × PBS and resuspended in 990 µL of 1 × PBS with 10 µL ribonuclease solution (Nippon Gene) to exclude RNA contamination, followed by the incubation for 30 min at 25 °C. Cellular DNA was stained by adding 50 µL of propidium iodide (1 µg/µL in PBS; Fujifilm Wako Pure Chemical) and incubated for 1 min at 25 °C in the dark. Flow cytometry analyses were performed using a Guava Easy Cyte Plus instrument (GE Healthcare) and FlowJo software (BD Biosciences).

### Apoptosis assay

The apoptosis assay was performed using the Caspase-Glo 3/7 Assay System (Promega Corporation, Madison, Wisconsin, USA). Briefly, 2.0 × 10^4^ cells were seeded into 96 well-white-walled plates with clear bottoms (Greiner Bio-One). After culturing for 24 h, 100 µL of Caspase-Glo Detection Solution was added to each well, and the plate was incubated for 1 h at 25 °C. The luminescence was measured using a Glomax 96 Microplate Luminometer (Promega).

### RNA sequencing

RNA sequencing was performed by Rhelixa Co. (Tokyo, Japan). BxPC-3 cells expressing TetOn shWWP1 were treated with or without 1 µg/mL of Dox and cultured for 48 h. Total RNA was extracted from the cells using the RNeasy Mini kit (QIAGEN), and the quality of the extracted RNA was assessed using a NanoDrop One spectrophotometer (Thermo Scientific) and Bioanalyzer (Agilent), ensuring an RNA integrity number (RIN) greater than 7. RNA libraries were prepared using the NEBNext Poly (A) mRNA Magnetic Isolation Module (#E7490, New England Biolabs (NEB), Ipswich, MA, USA) and the NEBNext UltraTM II Directional RNA Library Prep Kit (#E7760, NEB) according to the manufacturer's instructions. The prepared complementary DNA libraries were sequenced using a NovaSeq 6000 system (Illumina) to generate 150 base paired-end reads. Each sample generated approximately 8 G bases of data, corresponding to 53.3 million reads (26.7 million pairs) per sample. Quality control of the raw sequencing reads was performed using the Trimmomatic software (ver. 0.38) with the following settings: ILLUMINACLIP 2:30:10, LEADING = 20, TRAILING = 20; SLIDING WINDOW, 4:15; and MINLEN, 36. Low-quality reads and adapter sequences were also removed. The trimmed reads were then aligned to the reference genome (hg38) using the HISAT2 software (ver. 2.1.0). The transcript abundance was quantified using featureCounts (ver. 1.6.3) to calculate the raw read counts mapped to known exon regions, which were further normalized to Transcripts Per Million (TPM) values. Gene enrichment analyses were performed using GSEA. A false discovery rate (FDR) of less than 0.25 was considered statistically significant in the GSEA.

### Subcutaneous xenograft model

For xenograft transplantation, 2.0 × 10^5^ BxPC-3 cells expressing TetOn shWWP1 in 75 µL of RPMI were mixed with 75 µL of Matrigel TM-Growth Factor Reduced (#356231, Corning Incorporated, Corning, NY, USA), and immediately injected subcutaneously into the backs of male *BALB/cAjcl-Foxn1*^*nu*^ mice (CREA Japan Inc). The mice were maintained for 2 weeks to allow the transplanted cells to develop tumors and then divided into two groups with similar distribution of tumor sizes: one group received Dox at 1 mg/mL in a 5 mg/mL sucrose solution, and the other group did not receive Dox. The mice were then maintained for an additional 2 weeks, after which the resulting tumors were excised and weighed.

### Immunohistochemistry

Immunohistochemistry (IHC) was performed as previously described [28]. Briefly, fixed paraffin-embedded tissue microarrays T141c and PA805c (US Biomax, Derwood, MD, USA) were deparaffinized and incubated in Target Retrieval Solution (Dako Corp., Carpinteria, CA, USA) for 60 min at 98 °C for antigen retrieval. Endogenous peroxidase activity was blocked by incubation with 3% hydrogen peroxide for 10 min. After blocking in 10% normal goat serum (Dako) for 30 min, the sections were incubated overnight at 4 °C with a primary antibody diluted in 2% bovine serum albumin/PBS with 0.1% Tween-20. The sections were incubated for 30 min with a biotinylated secondary antibody (Vector Laboratories), washed, incubated for 30 min with an avidin–biotin–peroxidase complex (Vectarstain ABC Elite kit; Vector Laboratories), and developed by 3,3'-diaminobenzidine (DAB) substrate solution (DAKO Japan). Hematoxylin was used for nuclear staining. The primary antibodies, conditions, and dilutions used are listed in Supplementary Table S1.

### Inhibitor drug screening

Drug screening was performed using SCADS inhibitor kits (Kit I ver. 4, kit IV, ver. 2.4) obtained from the Molecular Profiling Committee (Ministry of Education, Culture, Sports, Science, and Technology, Japan). In addition, Buparlisib, Alpelisib, Taselisib, and TGX-221 (Selleck Chemicals, Houston, TX, USA) were included in the screening. Cells were plated at a seeding density of 100 cells/well in a 96-well plate (Greiner Bio-One) and incubated for 16 h. Subsequently, 178 drugs were dissolved in DMSO at the concentration of 100 µM and applied to the wells at a final concentration of 1 µM. Equal amounts of DMSO were added to the control wells. After 120 h of incubation, the number of cells in each well was examined using the CellTiter-Glo Assay System (Promega) according to the manufacturer’s instructions. Absorbance was measured using SpectraMax iD3 (Molecular Devices).

### Drug sensitivity assay

To determine the IC_50_ values of the drugs, 1.0 × 10^4^ BxPC-3 cells expressing TetOn shWWP1 were treated with doxycycline and stepwise concentrations of drugs for 120 h. Linsitinib and Torkinib were purchased from Selleck, and Indole-3-carbinol (I3C) was purchased from Millipore Sigma. Cell numbers after treatment with each drug were evaluated using a crystal violet assay. The absorbance of each sample was normalized to that of the control well, and dose–response curves were generated to determine the IC_50_ values. Drug synergism assessments were performed using the Combenefit Software (Cancer Research UK Cambridge Institute).

### Statistical analyses

The data are presented as mean ± standard deviation (SD) for each study group. For comparison between the two groups, the Student’s *t*-test or Mann–Whitney *U* test was used according to the data distribution. *χ*^2^ test or Fisher’s exact test was used to evaluate the immunohistochemistry of tissue microarrays. One-way analysis of variance (ANOVA) followed by the post-hoc Tukey–Kramer multiple comparison test was used for the apoptosis assay. Two-way ANOVA followed by the post-hoc Tukey–Kramer multiple comparison test was used for the cell proliferation assay and cell cycle analyses. Statistical significance was set at a *p*-value < 0.05. Data analysis was performed using GraphPad Prism software (version 10; Developer; San Diego, CA, USA).

## Results

### WWP1 expression was significantly upregulated in PDAC

**Fig. 1:**
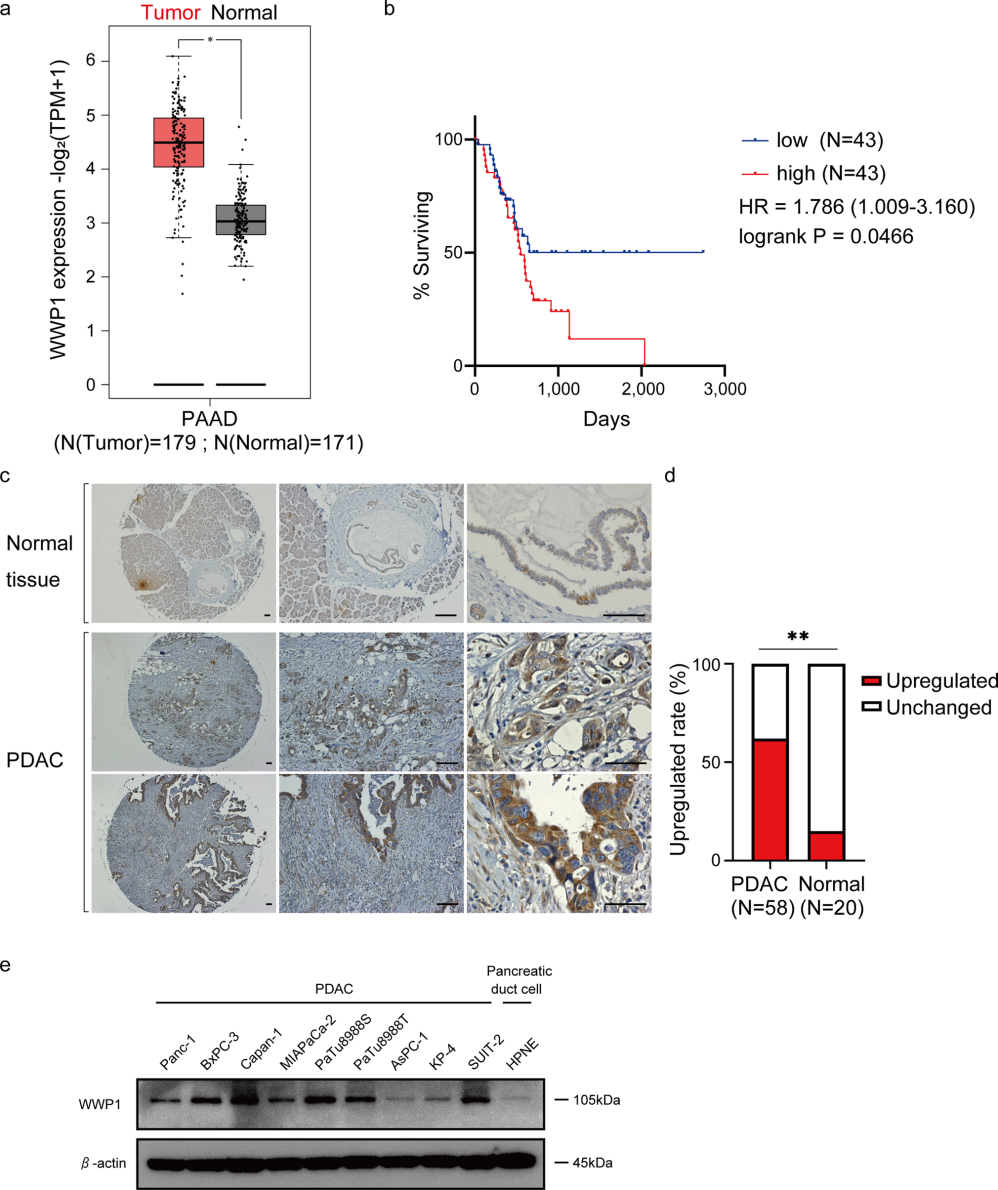
WWP1 expression was significantly upregulated in the PDAC tissues and cell lines. **a** WWP1 expression profile across pancreatic tumor samples and paired normal tissues according to the GEPIA database (http://gepia.cancer-pku.cn/). **b** Kaplan–Meier survival curve for patients with PDAC divided into *WWP1*-high or *WWP1*-low groups. Patient information was collected from The Cancer Genome Atlas database. **c** Representative images of immunohistochemistry for WWP1 in normal and PDAC tissues; scale bars = 50 µm. **d** Percentages of sections in which the WWP1 expression levels were elevated compared to the adjacent acinar regions. PDAC, *n* = 58 and normal pancreas, *n* = 20. Fisher’s exact test. **p < 0.01.** e** Western blot analyses show the expression profiles of WWP1 in multiple PDAC cell lines and an immortalized normal pancreatic ductal cell line. β-actin was used as the loading control

First, we investigated the *WWP1* expression signature in PDAC tissues compared to that in normal pancreatic tissues using data from the publicly available GEPIA database. The *WWP1* transcript levels were significantly elevated in pancreatic cancer tissues compared to those in normal pancreatic tissues (Fig. 1a). Furthermore, analyses using The Cancer Genome Atlas (TCGA) database revealed that the high *WWP1* expression signature in pancreatic cancer is associated with poor prognosis (Fig. 1b). To further evaluate the *WWP1* expression profile in the pancreatic tissues, we performed immunohistochemistry using a tissue microarray. WWP1 was intensely stained in the PDAC cells compared to that in normal pancreatic ductal cells (Fig. 1c). The proportion of specimens with increased staining intensity compared to the adjacent acinar tissue was significantly higher in the pancreatic cancer tissues than that in the normal pancreatic ductal tissues (Fig. 1d). We subsequently assessed the WWP1 expression pattern in PDAC cell lines and an immortalized pancreatic ductal cell line. Consistent with the immunohistochemical staining findings, the WWP1 expression levels were higher in most PDAC cells compared to those in HPNE cells (Fig. 1e).

### *WWP1* depletion suppressed the proliferation of PDAC cell lines

**Fig. 2:**
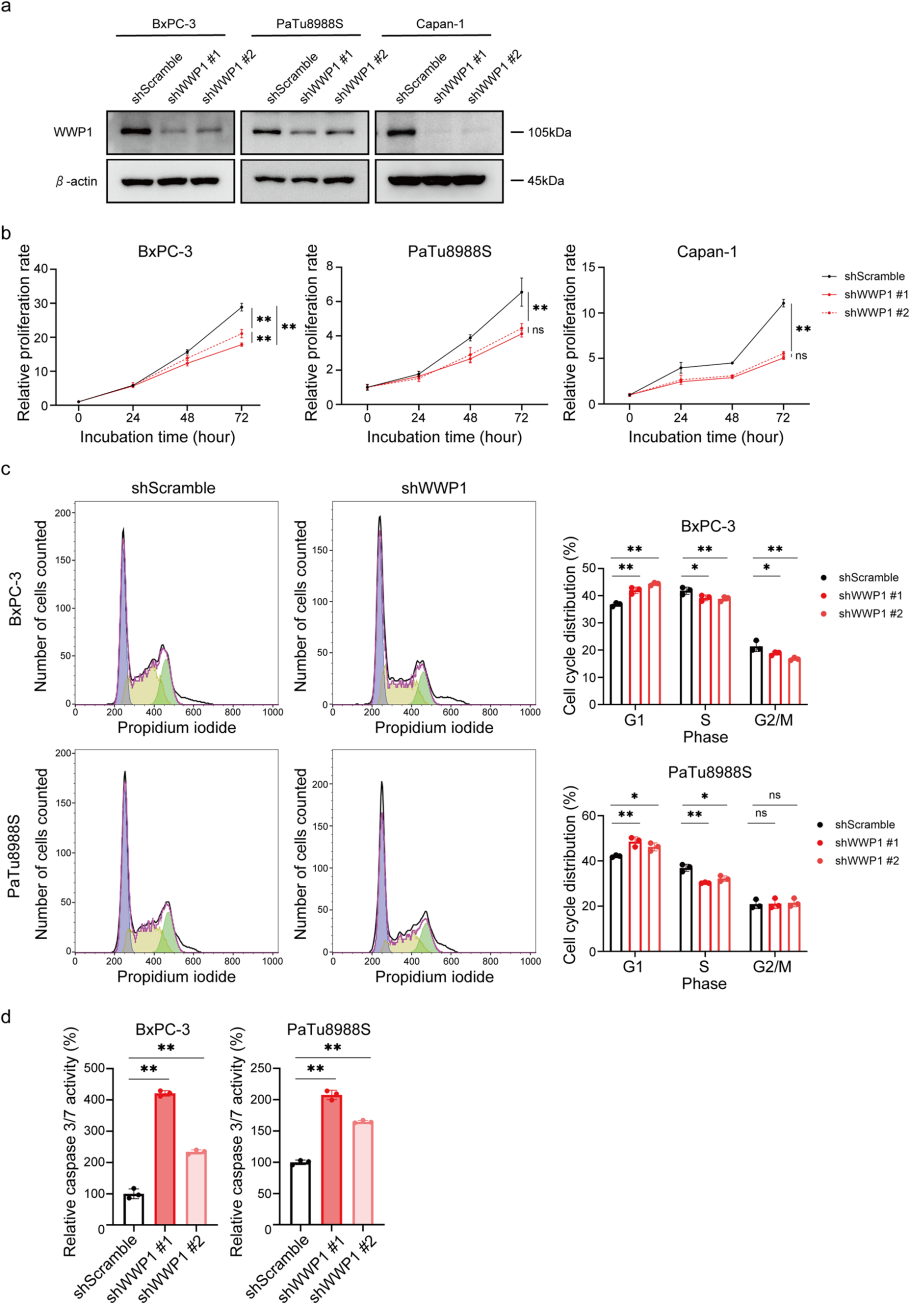
*WWP1* depletion suppressed the proliferation of PDAC cells. **a** Western blot analyses for the confirmation of *WWP1* knockdown in BxPC-3, PaTu8988S, and Capan-1 cells compared to that in shScramble-transfected cells. β-actin was used as the loading control.** b** Relative proliferation rate in BxPC-3, PaTu8988S, and Capan-1 shScramble and shWWP1 cells, quantified by crystal violet assay. Data are shown as mean ± SD of triplicate assays. Repeated-measured two-way ANOVA followed by post-hoc Tukey–Kramer multiple comparison test. **p < 0.01. **c** (left) Representative histograms of cell cycle analyses of BxPC-3 and PaTu8988S shScramble and shWWP1 cells. (right) Bar plots showing the percentage of cells in each of the cell cycle phases (G_0_/G_1_, S, G_2_/M). Data are shown as mean ± SD of triplicate assays. *p < 0.05, **p < 0.01. **d** Relative caspase 3/7 activity of shScramble and shWWP1 cells. Data are presented as mean ± SD. *p < 0.05, **p < 0.01. One-way ANOVA followed by post-hoc Tukey–Kramer multiple comparison test of triplicated assays

Next, we investigated the biological effects of WWP1 inhibition on PDAC cells to elucidate its potential role as a therapeutic target in PDAC. BxPC-3, PaTu8988S, and Capan-1 cells which exhibit markedly elevated WWP1 expression signature were selected for further analysis (Fig. 1e). For this purpose, knockdown cells that are stably expressing WWP1 shRNA were established (Fig. 2a). The *WWP1* knockdown cells exhibited a significant decrease in proliferation compared to the control cells in all the respective cell lines (Fig. 2b). Cell cycle analysis revealed a higher proportion of cells in the G1 phase in the *WWP1* knockdown cells than that in the control cells in these cell lines; this indicates that cell cycle arrest in the G1 phase was induced by *WWP1* depletion (Fig. 2c and S1a). Additionally, a significant increase in caspase 3/7 activity was observed in the *WWP1*-depleted BxPC-3, PaTu8988S, and Capan-1 cells compared to that in the control cells (Fig. 2d and S1b), which indicates that WWP1 is involved in the proliferative capacity of PDAC cells.

### *WWP1* depletion inhibits the PI3K-AKT pathway by influencing PTEN activity

**Fig. 3:**
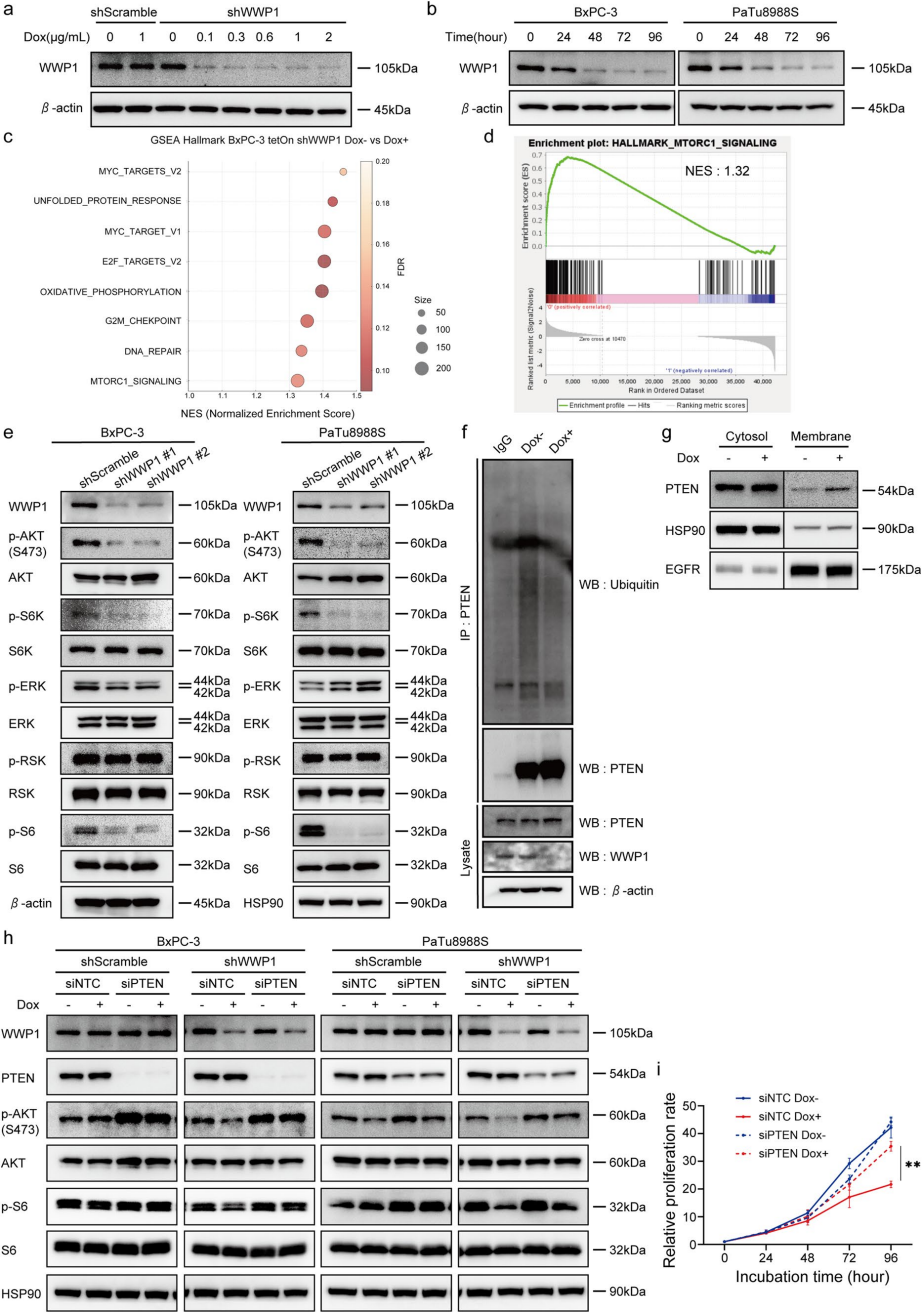
*WWP1* depletion suppressed the PI3K-AKT pathway through PTEN function. **a** Western blot analyses showing dose-dependent reduction of WWP1 expression in BxPC-3 cells with Dox-inducible shWWP1 expressing vector (BxPC-3 TetOn-shWWP1). Cells were treated with Dox for 48 h.** b** Western blotting images demonstrating the knockdown efficiency of WWP1 over time after the treatment with Dox (1 µg/mL). β-actin was used as the loading control.** c** GSEA analyses using MSigDB Hallmark gene sets (version 4.2.1), comparing *WWP1* knockdown (Dox-On) to control (Dox-Off) settings in BxPC-3 TetOn-shWWP1 cells. Only Gene sets with a false discovery rate (FDR) below 0.25 are shown.** d** The enrichment plot shows that the mTORC1 signaling pathway was positively enriched in the *WWP1* knockdown cells. **e** Western blot analyses of WWP1 and PI3K-AKT pathway proteins in BxPC-3 and PaTu8988S cells. HSP90 was used as the loading control. **f** Dox-treated and untreated BxPC-3 cells subjected to immunoprecipitation (IP) using an anti-PTEN antibody. Isotype control IgG was used as a negative control. **g** Western blot analysis of cytosolic and membrane fractions isolated from BxPC-3 TetOn-shWWP1 cells with or without Dox treatment. HSP90 and EGFR were used as markers for cytosolic and membrane fractions, respectively. Cells were harvested 48 h post-treatment.** h** Western blot analyses of WWP1 and PI3K-AKT pathway proteins. siPTEN and control siNTC were introduced into TetOn-shWWP1 cells with and without Dox treatment. Cells were harvested 48 h post-treatment. NTC, non-targeting control. **i** Cell proliferation assay of BxPC-3 cells with control, *WWP1* knockdown, and double knockdown of *WWP1* and *PTEN*. Data are presented as mean ± SD. Repeated-measured two-way ANOVA followed by post-hoc Tukey–Kramer multiple comparison test of triplicate assays. **p < 0.01

To enhance reproducibility, we introduced a doxycycline (Dox)-inducible shWWP1 expression vector (TetOn shWWP1) into the BxPC-3 and PaTu8988S cells. The expression of WWP1 was sufficiently repressed by Dox treatment (concentration, 0.1 µg/mL) after 48 h (Fig. 3a, b). These cells showed reduced proliferation by Dox-induced *WWP1* knockdown (Fig. S2). RNA sequencing was conducted to achieve a comprehensive analysis of the BxPC-3 TetOn shWWP1 cells and explore the mechanism underlying the reduced proliferative capacity of *WWP1* knockdown cells. GSEA database analysis revealed that the gene sets associated with proliferation capacity were enriched in the untreated BxPC-3 cells (Fig. 3c and S3), especially the mTORC1 signaling, a key component of the PI3K-AKT signaling pathway (Fig. 3d). Based on these results, we confirmed that the phosphorylation of AKT and S6 was suppressed in the *WWP1* knockdown cells, reflecting a reduction in the PI3K-AKT pathway (Fig. 3e). Previous reports have suggested that WWP1 decreases the PTEN activity, which in turn deregulates the activity of the PI3K-AKT pathway in prostate and breast cancer cells [16, 26]. Therefore, we investigated whether WWP1 ubiquitinates PTEN in PDAC cells. Immunoprecipitation analyses using an anti-PTEN antibody revealed that PTEN ubiquitination was reduced in BxPC-3 cells with *WWP1* knockdown (Fig. 3f). Additionally, the membrane fraction of PTEN was increased in *WWP1*-depleted cells (Fig. 3g), consistent with observations in prostate and breast cancer cells [16, 26]. These findings indicate that PTEN is a target substrate for WWP1 in PDAC cells. To evaluate whether the suppression of the PI3K-AKT pathway by *WWP1* depletion was PTEN-dependent, we introduced siRNAs targeting *PTEN* into the *WWP1* knockdown cells. We found that the reduction in phosphorylated AKT and S6 levels following *WWP1* knockdown was canceled by siPTEN (Fig. 3h). Moreover, PTEN expression was not altered by *WWP1* knockdown, which is consistent with a previous study [16]. Furthermore, the suppression of cell proliferation in the *WWP1* knockdown cells was restored when *PTEN* was also knocked down (Fig. 3i). These findings indicate that the reduced proliferative capacity of *WWP1*-depleted cells is mediated by the inhibition of the PI3K-AKT pathway through PTEN activation.

### WWP1 is involved in the activation of the PI3K-AKT pathway in PDAC tissues

**Fig. 4:**
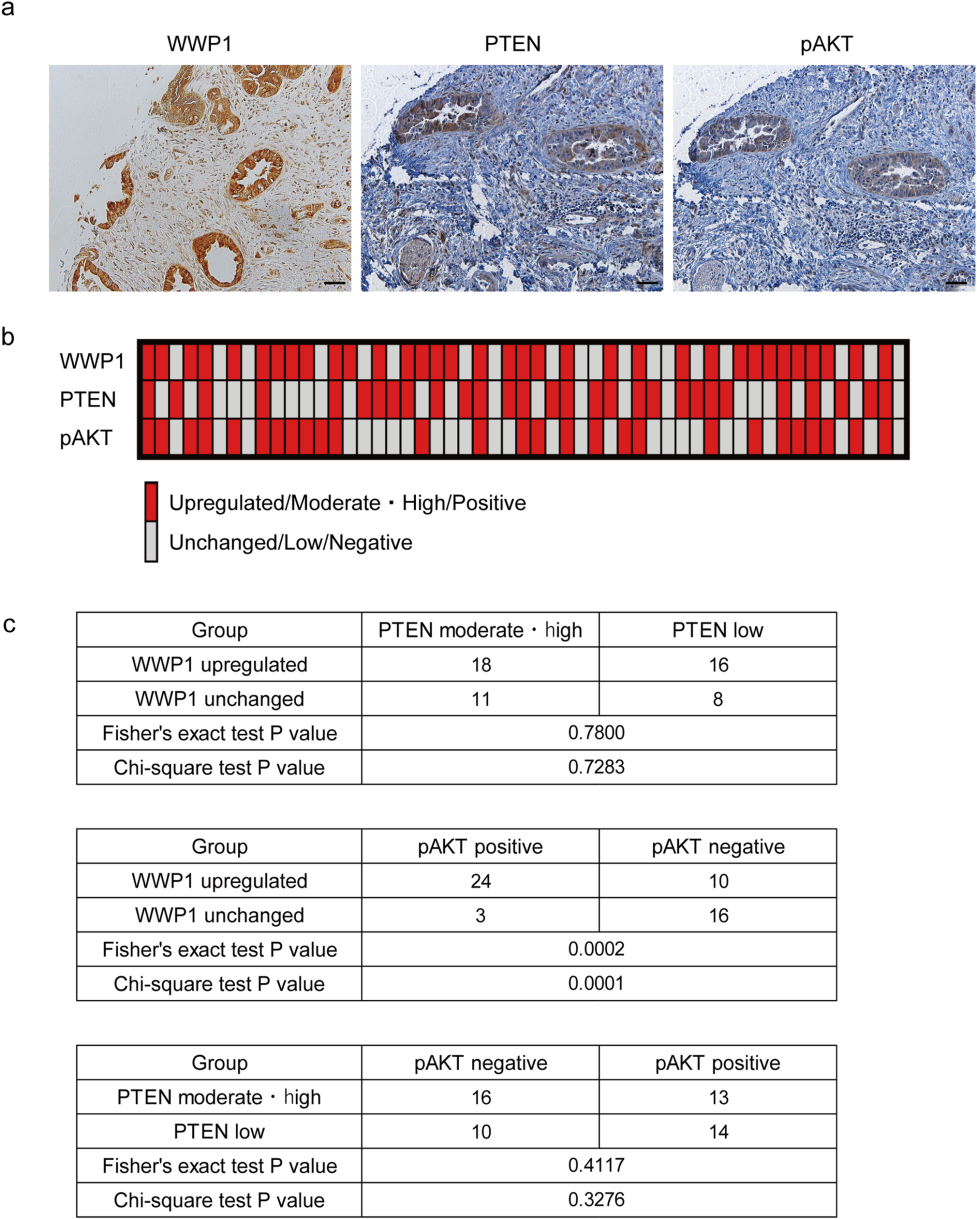
WWP1 expression was correlated to the activity of the PI3K-AKT pathway in clinical specimens. **a** Representative immunohistochemistry images of WWP1, PTEN, and pAKT staining of the tissue microarray. Each panel represents a section with upregulated WWP1, moderate/high PTEN, and positive pAKT, respectively. Scale bar: 50 µm. **b** Binary heatmap representing the expression pattern of WWP1, PTEN, and pAKT in each section. **c** Statistical analyses of the correlation between WWP1, PTEN, and pAKT. The Fisher’s exact test and χ^2^ test were performed for each comparison

To confirm the relationship between the WWP1 and PTEN expression levels and activity of the PI3K-AKT pathway in human specimens, we performed immunohistochemistry using tissue microarrays from 53 cases (Fig. 4a and S4). WWP1 was upregulated in 34 cases, PTEN expression was decreased in 24 cases, and pAKT expression was positive in 27 cases, respectively (Fig. 4b). Correlation analyses revealed a significant correlation between WWP1 upregulation and pAKT positivity, whereas no significant relationship was observed between WWP1 upregulation and PTEN reduction, nor between PTEN expression and pAKT positivity (Fig. 4c). These findings suggest that WWP1 upregulation contributes to PI3K-AKT pathway activation in PDAC tissues by impairing PTEN function rather than downregulating PTEN expression.

### *WWP1* depletion suppresses tumor growth in vivo by inhibiting the PI3K-AKT pathway

**Fig. 5:**
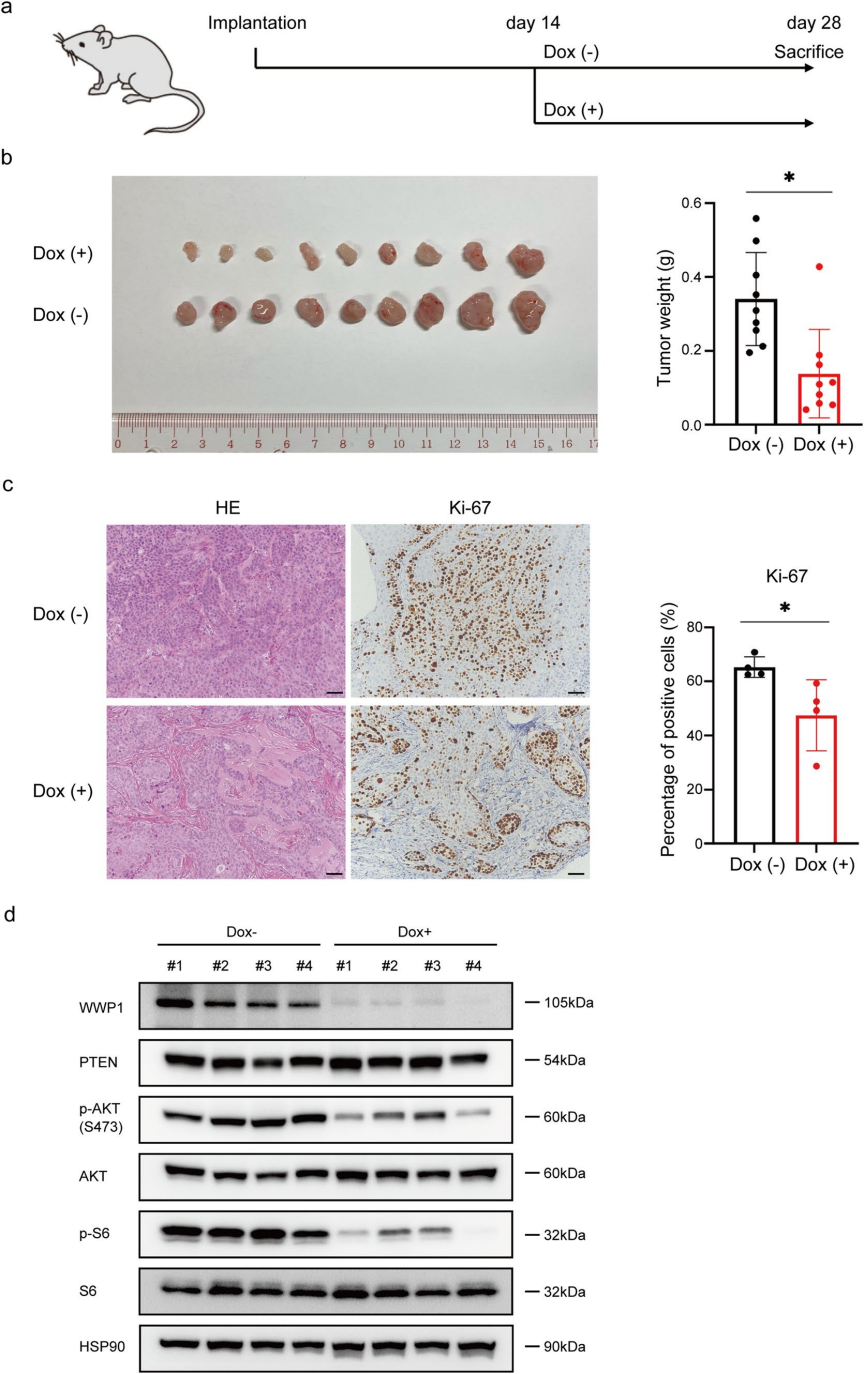
*WWP1* depletion significantly suppressed the tumor growth in a xenograft mouse model. **a** Scheme of the xenograft assay study design.** b** Images of dissected tumors and tumor weight in Dox-treated and untreated groups. Data are presented as mean ± SD. Student’s *t*-test. *p < 0.05.** c** (left) Representative images of H&E staining and immunohistochemistry for Ki-67; scale bars = 50 µm. (right) Percentage of Ki-67 positive tumor cells. Data are shown as mean ± SD. Student’s *t*-test. *p < 0.05. **d** Western blot analysis of the xenograft tumors. HSP90 was used as a loading control

To evaluate the anti-tumor effects of WWP1 inhibition in vivo, we conducted a xenograft assay using subcutaneously transplanted PDAC cells in mice. Two weeks after the TetOn shWWP1 BxPC-3 cells were subcutaneously transplanted, the mice were divided into two groups with similar distribution of tumor sizes. They were then administrated sugar water with or without Dox for 2 additional weeks (Fig. 5a). Dissected tumors in the *WWP1* knockdown group were significantly smaller than those in the control group (Fig. 5b). Consistently, immunohistochemical analysis of Ki-67 expression showed a decreased positivity rate in the *WWP1* knockdown tumors (Fig. 5c). Western blotting analysis confirmed the PI3K-AKT pathway suppression in the *WWP1* knockdown tumors (Fig. 5d). These findings demonstrated that *WWP1* depletion inhibited tumor growth and activation of the PI3K-AKT pathway in vivo.

### WWP1 inhibition enhances the effect of PI3K-AKT pathway inhibitors

**Fig. 6:**
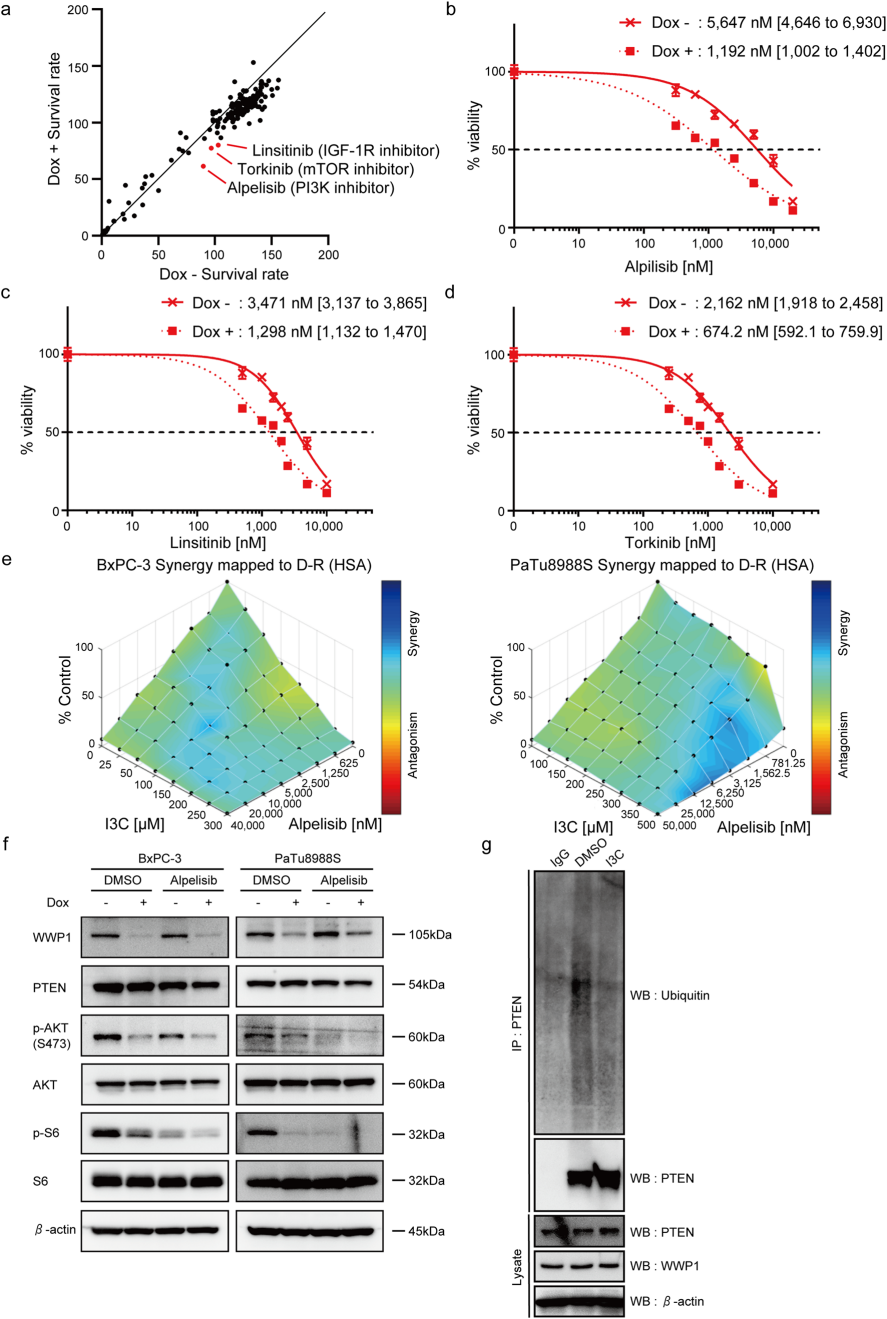
WWP1 inhibition enhanced the effectiveness of PI3K-AKT pathway inhibitors. **a** Dot plot of the cell survival rates from the drug screening test with BxPC-3 TetOn-shWWP1 cells with and without Dox treatment. Each dot represents a screened compound. alpelisib, linsitinib, and torkinib are shown in red dots. **b–d** Dose–response curves for alpelisib, linsitinib, and torkinib in BxPC-3 cells with and without *WWP1* knockdown. **e** 3D synergy score plots for combinatory treatment with I3C and alpelisib in BxPC-3 and PaTu8988S cells. The synergic performance score was analyzed using Combenefit software (highest single agent (HSA) method). **f** Western blot analysis of BxPC-3 and PaTu8988S cells with *WWP1* knockdown treated with alpelisib. Alpelisib was administered at 1µM simultaneously with Dox; the cells were harvested 48 h post-treatment. **g** Western blot analysis of PTEN ubiquitination. BxPC-3 cells were treated with 100 µM I3C for 48 h or left untreated and subjected to IP using an anti-PTEN antibody. Isotype control IgG was used as a negative control

To explore the clinical significance of WWP1 inhibition, drug screening assays were conducted to identify potential drugs that exhibit additive or synergistic effects when combined with *WWP1* depletion (Table S2). Linsitinib, an IGF-1R inhibitor; torkinib, an mTOR inhibitor; and alpelisib, a PI3K inhibitor, decreased the survival rate of BxPC-3 cells when combined with *WWP1* knockdown (Fig. 6a). This was further validated by the significant reduction in the IC50 value of each drug upon *WWP1* knockdown in BxPC-3 cells (Fig. 6b–d). All three compounds target the PI3K-AKT pathway; thus, WWP1 depletion may act as an additional inhibitor, leading to additive effects when combined with other inhibitors that target the same pathway. We further investigated the effects of combination therapy with pharmacological inhibition of WWP1 and PI3K-AKT pathway inhibitors. Combination treatment with Indole-3-carbinol (I3C), previously identified as an inhibitor of the ubiquitin ligase activity of WWP1 [16], and alpelisib exhibited additive inhibitory effects in BxPC-3 and PaTu8988S cells (Fig. 6e). Assessment of the PI3K-AKT pathway signaling after *WWP1* knockdown combined with alpelisib treatment revealed a marked reduction in the phosphorylation of AKT and S6 (Fig. 6f). Additionally, I3C treatment was shown to reduce PTEN ubiquitination (Fig. 6g), whereas the therapeutic effect of I3C was abrogated by *WWP1* knockdown (Fig. S5). These findings suggest that the primary mechanism of I3C in pancreatic cancer cells is mediated through the WWP1-PTEN axis. These findings suggest that WWP1 inhibition potentiates the effect of PI3K-AKT pathway inhibitors through activating PTEN.

## Discussion

In this study, we demonstrated that WWP1 is upregulated in PDAC tissues. Moreover, *WWP1* depletion inhibited the cellular proliferation, particularly in the cell lines that have high WWP1 expression levels, namely BxPC-3, PaTu8988S, and Capan-1. Considering that WWP1 functions as a ubiquitin ligase that targets multiple cancer-related genes, we conducted a comprehensive analysis using RNA sequencing and identified its association with the PI3K-AKT pathway in PDAC cells. Consistent with previous reports on breast, prostate, and colon cancers [16, 17, 26], PTEN was identified as a target of WWP1 in PDAC cell lines in which the inhibition of proliferation by *WWP1* knockdown was largely reversed by *PTEN* silencing. Importantly, the ubiquitination of PTEN by WWP1 disrupts its dimerization and causes its dissociation from the plasma membrane, impairing its function as a dephosphorylating enzyme without degradation via the ubiquitin–proteasome machinery [16, 29]. This suggests that PTEN dysfunction can occur in PTEN-intact tumors when WWP1 expression or activity is upregulated, which may not be detected by genomic or transcriptomic analyses. Since the incidence of *PTEN* mutations or deletions is rare in PDAC, the therapeutic significance of targeting PTEN function may be underestimated. Indeed, clinical samples revealed a significant number of patients with elevated WWP1 expression and positive pAKT staining without a reduction in PTEN expression.

Notably, a drug-screening assay demonstrated that several PI3K-AKT pathway inhibitors exhibited additive effects with WWP1 inhibition. Currently, there are no approved PI3K-AKT pathway inhibitors for the treatment of PDAC [30–32]. This limitation is primarily due to the rapid development of drug resistance and severe side effects. One of the main mechanisms of resistance is the decreased expression or deletion of *PTEN* [33, 34]. Dual inhibition of WWP1 and PI3K has shown a synergistic growth-inhibitory effect in breast cancer cells that have acquired resistance to PI3K inhibitors, provided that PTEN is not completely deleted. This suggests that WWP1 inhibition can reactivate PTEN function, even when its expression is suppressed, thereby contributing to further suppression of the PI3K/AKT pathway and potentially mitigating negative feedback loops associated with resistance [26]. Although further investigation is required, WWP1 inhibitors hold promise for enhancing PI3K-AKT pathway suppression in pancreatic cancer, while simultaneously reducing the risk of resistance.

The mechanisms that regulate the expression levels of WWP1 in PDAC require further elucidation. Previous reports have demonstrated that the proto-oncogene *MYC* functions as a promotor for the transcription of *WWP1* [16, 26]. MYC amplification, which is associated with poor prognosis and metastasis in pancreatic cancer [35, 36], frequently co-occurs with WWP1 amplification. Further research is essential to identify the subclasses of pancreatic cancers that exhibit high WWP1 expression signatures. This provides critical insights for predicting the anti-tumor effects of WWP1 inhibition in combination with PI3K-AKT pathway inhibitors, potentially improving the prognosis of PDAC.

## Supplementary Information

Below is the link to the electronic supplementary material.

Supplementary file1 (PDF 74167 KB)

Supplementary file2 (XLSX 10 KB)

Supplementary file3 (XLSX 16 KB)
