# Supplementary material for: WWP1 inhibition suppresses the proliferation of pancreatic cancer cells by regulating the PI3K-AKT pathway
Source: J Gastroenterol. 2024 Dec 10;60(3):370–84. doi: 10.1007/s00535-024-02192-x (PMC11880106; doi:10.1007/s00535-024-02192-x)
Supplement: Supplementary file 1 — Supplementary file1 (PDF 74167 KB) [file 535_2024_2192_MOESM1_ESM.pdf]

Supplementary Information

WWP1 inhibition suppresses the proliferation of pancreatic cancer cells by regulating the PI3K-AKT pathway

Journal of Gastroenterology

Genso Notoya[1,2], Takahiro Kishikawa[2]*, Kengo Yasugi[2], Takuma Iwata[2], Takahiro Seimiya[2], Koji Miyabayashi[2], Ryota Takahashi[2], Keisuke Yamamoto[2], Hideaki Ijichi[2,3], Motoyuki Otsuka[2], and Mitsuhiro Fujishiro[2]

[1]Department of Endoscopy and Endoscopic Surgery, Graduate School of Medicine, The University of Tokyo, Tokyo, 113-8655, Japan

[2]Department of Gastroenterology, Graduate School of Medicine, The University of Tokyo, Tokyo, 113-8655, Japan

[3]Clinical Nutrition Center, Graduate School of Medicine, The University of Tokyo, Tokyo, 113-8655, Japan

*Corresponding author:

Takahiro Kishikawa M.D., Ph.D.,

Department of Gastroenterology, Graduate School of Medicine, The University of Tokyo, 7-3-1 Hongo, Bunkyo-ku, Tokyo 113-8655, Japan.

Phone number: +81-3-3815-5411; Extension: 30200

Fax number: +81-3-3814-0021

E-mail address: tkishikawa-tky@g.ecc.u-tokyo.ac.jp

Supplementary Figures and legends (Figures S1-S5)

Fig. S1. *WWP1* depletion induces cell cycle arrest and apoptosis in Capan-1 cells.

Fig. S2. Dox-inducible *WWP1* depletion suppresses the proliferation of PDAC cells.

Fig. S3. Gene sets associated with proliferation capacity were downregulated in *WWP1*-depleted cells.

Fig. S4. Immunohistochemical staining categories for WWP1, PTEN, and pAKT.

Fig. S5. Dose-response curve for I3C in *WWP1* knockdown cells.

Supplementary Tables (Table S1-S2)

Table S1. List of antibodies used in this study.

Table S2. List of drugs used for the drug screening assay.

a

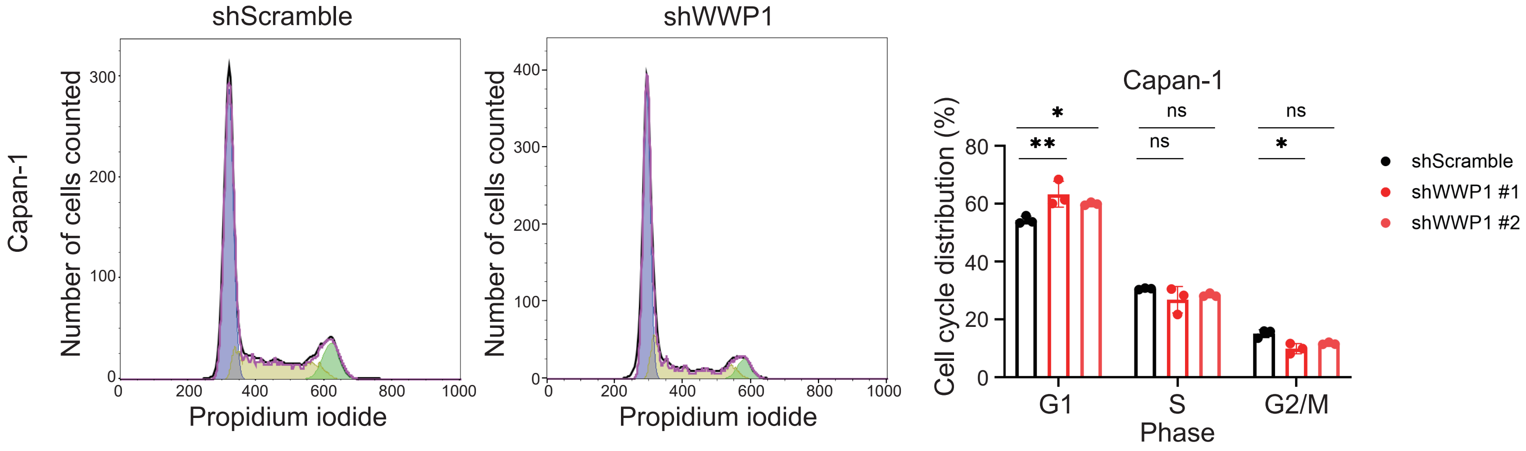
Capan-1
shScramble
shWWP1
Number of cells counted
Propidium iodide
0
200
400
600
800
1000
100
200
300
400
Capan-1
Cell cycle distribution (%)
0
20
40
60
80
G1
S
G2/M
Phase
*
**
ns
ns
ns
*
shScramble
shWWP1 #1
shWWP1 #2

b

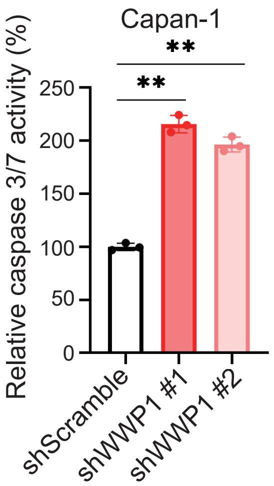
Capan-1
Relative caspase 3/7 activity (%)
0
50
100
150
200
250
**
**
shScramble
shWWP1 #1
shWWP1 #2

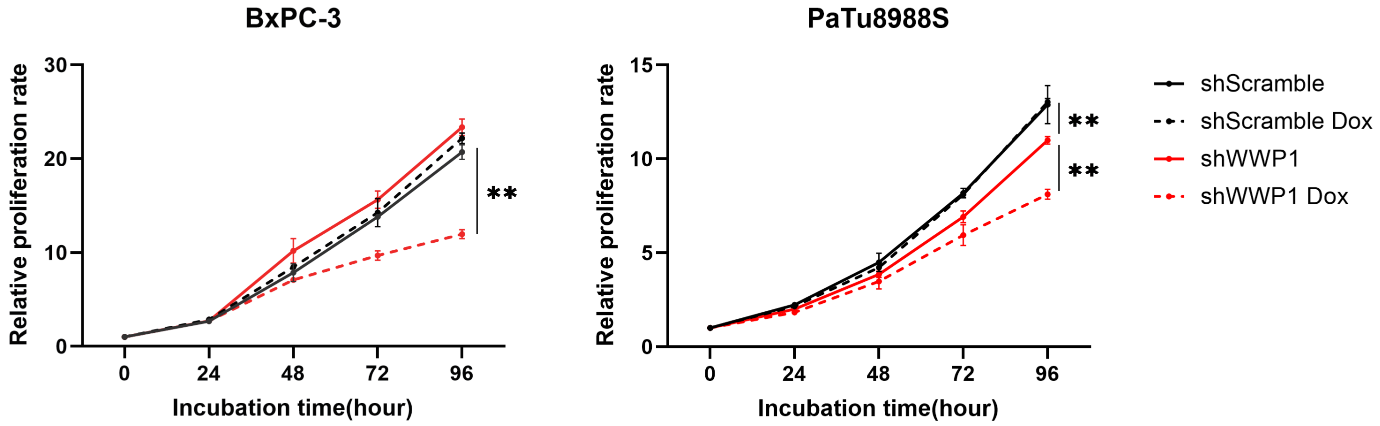
BxPC-3
Relative proliferation rate
30
20
10
0
0
24
48
72
96
Incubation time(hour)
**
PaTu8988S
Relative proliferation rate
15
10
5
0
0
24
48
72
96
Incubation time(hour)
**
**
shScramble
shScramble Dox
shWWP1
shWWP1 Dox

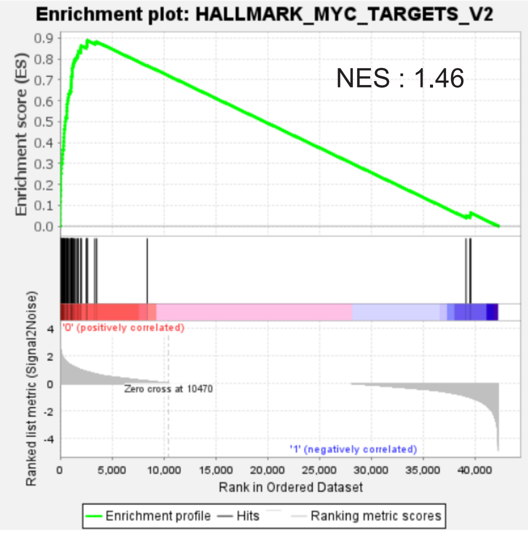
Enrichment plot: HALLMARK_MYC_TARGETS_V2
NES : 1.46
Enrichment score (ES)
Ranked list metric (Signal2Noise)
'0' (positively correlated)
Zero cross at 10470
'1' (negatively correlated)
Rank in Ordered Dataset
Enrichment profile
Hits
Ranking metric scores

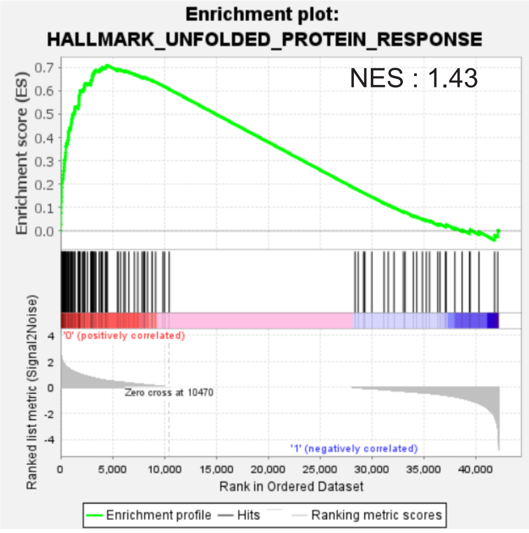
Enrichment plot:
HALLMARK_UNFOLDED_PROTEIN_RESPONSE
NES : 1.43
Enrichment score (ES)
Ranked list metric (Signal2Noise)
'0' (positively correlated)
Zero cross at 10470
'1' (negatively correlated)
Rank in Ordered Dataset
Enrichment profile
Hits
Ranking metric scores

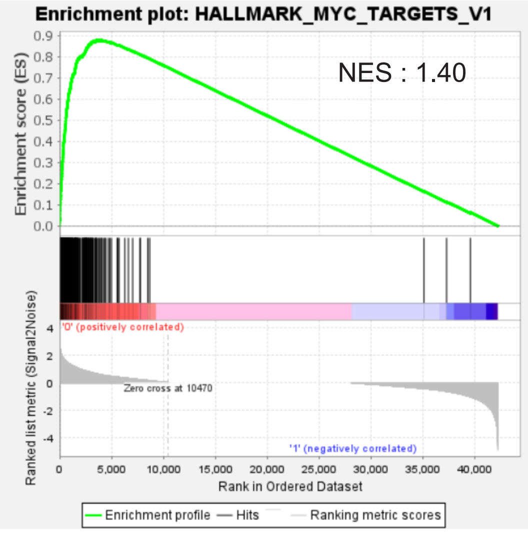
Enrichment plot: HALLMARK_MYC_TARGETS_V1
NES : 1.40
Enrichment score (ES)
Ranked list metric (Signal2Noise)
'0' (positively correlated)
Zero cross at 10470
'1' (negatively correlated)
Rank in Ordered Dataset
Enrichment profile
Hits
Ranking metric scores

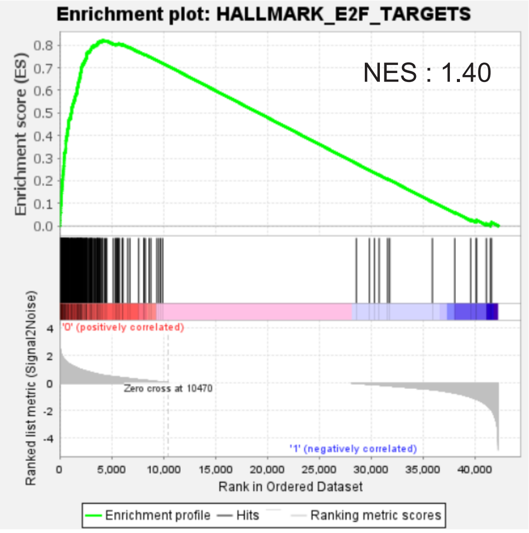
Enrichment plot: HALLMARK_E2F_TARGETS
NES : 1.40
Enrichment score (ES)
Ranked list metric (Signal2Noise)
'0' (positively correlated)
Zero cross at 10470
'1' (negatively correlated)
Rank in Ordered Dataset
Enrichment profile
Hits
Ranking metric scores

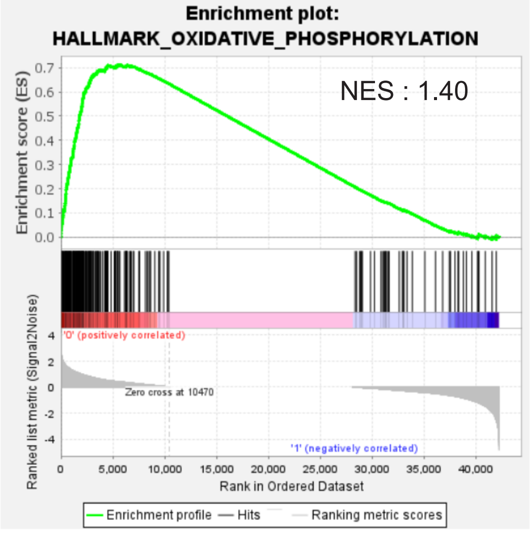
Enrichment plot:
HALLMARK_OXIDATIVE_PHOSPHORYLATION
NES : 1.40
Enrichment score (ES)
Ranked list metric (Signal2Noise)
'0' (positively correlated)
Zero cross at 10470
'1' (negatively correlated)
Rank in Ordered Dataset
Enrichment profile
Hits
Ranking metric scores

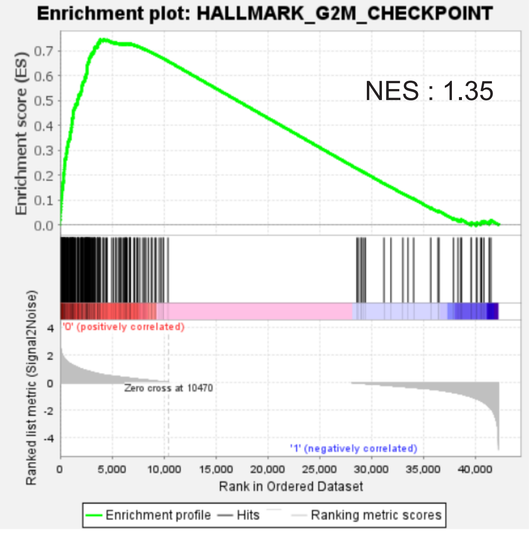
Enrichment plot: HALLMARK_G2M_CHECKPOINT
NES : 1.35
Enrichment score (ES)
Ranked list metric (Signal2Noise)
'0' (positively correlated)
Zero cross at 10470
'1' (negatively correlated)
Rank in Ordered Dataset
Enrichment profile
Hits
Ranking metric scores

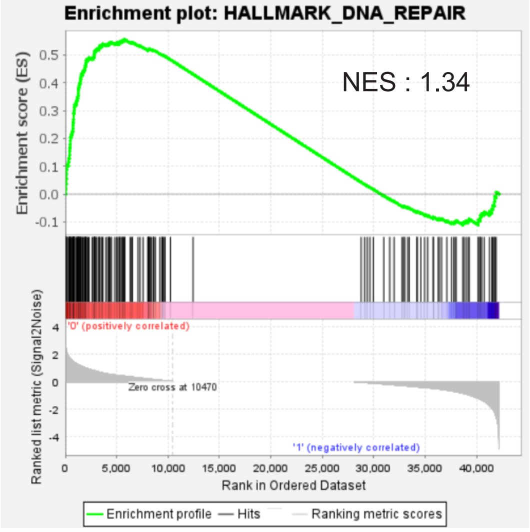
Enrichment plot: HALLMARK_DNA_REPAIR
NES : 1.34
Enrichment score (ES)
Ranked list metric (Signal2Noise)
'0' (positively correlated)
Zero cross at 10470
'1' (negatively correlated)
Rank in Ordered Dataset
Enrichment profile
Hits
Ranking metric scores

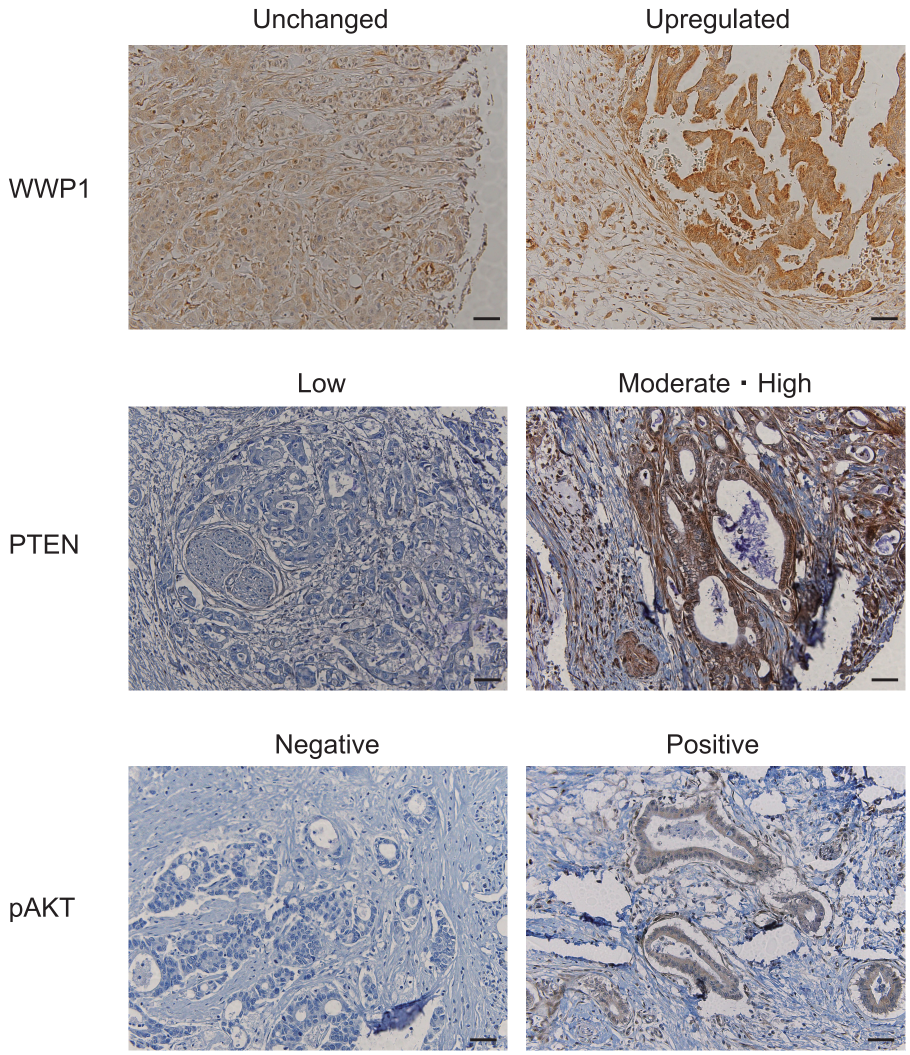
Unchanged
Upregulated
WWP1
Low
Moderate・High
PTEN
Negative
Positive
pAKT

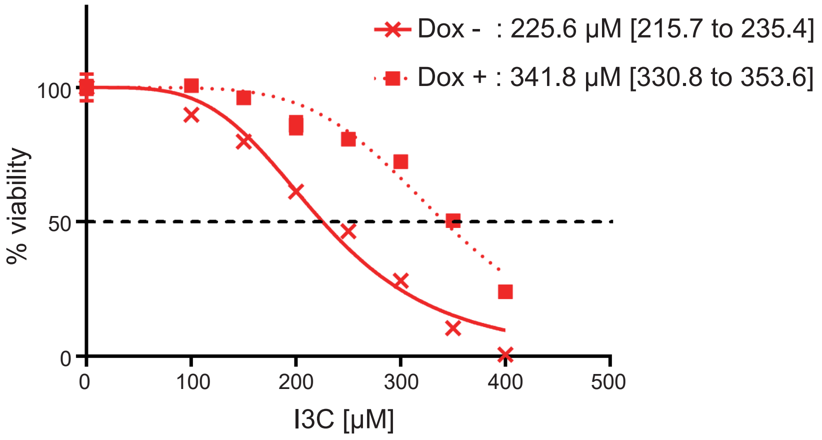

Dox - : 225.6 μM [215.7 to 235.4]
Dox + : 341.8 μM [330.8 to 353.6]
% viability
100
50
0
0
100
200
300
400
500
I3C [μM]

Supplementary Figure Legends

Figure S1. *WWP1* depletion induces cell cycle arrest and apoptosis in Capan-1 cells.

(a) (Left) Representative histograms of cell cycle analysis in Capan-1 shScramble and shWWP1 cells. (Right) Bar plots showing the percentage of cells in each cell cycle phase (G_0/G_1, S, G_2/M). Data are presented as mean ± SD from triplicate assays. *$p < 0.05$, **$p < 0.01$. **(b)** Relative caspase 3/7 activity in Capan-1 shScramble and shWWP1 cells. Data are shown as mean ± SD from triplicate assays. **$p < 0.01$. Statistical analysis was performed using one-way ANOVA followed by the post-hoc Tukey–Kramer multiple comparison tests.

Figure S2. Dox-inducible *WWP1* depletion suppresses the proliferation of PDAC cells.

Relative proliferation rate in BxPC-3 and PaTu8988S control and TetOn-shWWP1 cells, with and without Dox, quantified by crystal violet assay. Data are shown as mean ± SD of triplicate assays. Repeated-measured 2-way ANOVA followed by Tukey-Kramer multiple comparison test. **$p < 0.01$.

Figure S3. Gene sets associated with proliferation capacity were downregulated in

***WWP1*-depleted cells.**

Enrichment plots of the gene sets significantly enriched in *WWP1*-depleted BxPC-3 cells with a false discovery rate (FDR) below 0.25.

Figure S4. Immunohistochemical staining categories for WWP1, PTEN, and pAKT.

Representative images categorizing the staining intensity of WWP1, PTEN, and pAKT into two groups based on IHC. Scale bar = 50 μm.

Figure S5. Dose-response curve for I3C in WWP1 knockdown cells.

Dose-response curves for I3C in BxPC-3 TetON shWWP1 cells with and without Dox treatment.
